# Proteomic Analysis of Serum in Cardiac Transthyretin Amyloidosis: Diagnostic and Prognostic Implications for Biomarker Discovery

**DOI:** 10.3390/biomedicines13071647

**Published:** 2025-07-06

**Authors:** Joanna Waś, Monika Gawor-Prokopczyk, Agnieszka Sioma, Rafał Szewczyk, Aleksandra Pel, Jolanta Krzysztoń-Russjan, Magdalena Niedolistek, Dorota Sokołowska, Jacek Grzybowski, Łukasz Mazurkiewicz

**Affiliations:** 1 Department of Medical Biology, National Institute of Cardiology, State Research Institute, 42 Alpejska Str., 04-628 Warsaw, Poland; jkrzyszton@ikard.pl (J.K.-R.); mniedolistek@ikard.pl (M.N.); dsokolowska@ikard.pl (D.S.); 2 Department of Cardiomyopathy, National Institute of Cardiology, State Research Institute, 42 Alpejska Str., 04-628 Warsaw, Poland; asioma@ikard.pl (A.S.); jgrzybowski@ikard.pl (J.G.); lmazurkiewicz@ikard.pl (Ł.M.); 3 LabExperts sp z o.o., 7A Limbowa Str., 80-175 Gdansk, Poland; rafal.szewczyk@labexperts.com.pl (R.S.); aleksandra.pel@labexperts.com.pl (A.P.)

**Keywords:** transthyretin amyloid cardiomyopathy, cardiac amyloidosis, transthyretin, transthyretin amyloidosis

## Abstract

**Background/Objectives:** Having serum biomarkers available for cardiac transthyretin amyloidosis (ATTR-CA) would be beneficial for diagnosis and prognosis. This study aimed to identify potential ATTR-CA biomarkers through proteomic analysis. **Patients and Methods:** Serum proteomic analyses were conducted on 15 ATTR-CA patients before receiving treatment, 11 ATTR-CA patients who had received tafamidis treatment for at least six months, and 13 patients with suspected cardiac amyloidosis who were later ruled out. All patients underwent blood tests, standard 12-lead electrocardiography, transthoracic echocardiography, and ^99m^Tc-DPD scintigraphy. **Results:** Proteomic analysis revealed significant differences in protein levels among the study groups. Key findings revealed increased levels of several proteins, including ceruloplasmin, apolipoprotein E, SERPINA1, and cDNA FLJ54111 (which is highly similar to serum transferrin), in ATTR-CA patients before receiving specific treatment. There was also a reduction in prothrombin, transferrin, CD14, and alpha-2-macroglobulin. In the ATTR-CA group treated with tafamidis, elevated levels of SERPINA1, paraoxonase 1, and complement C2 were observed. Notably, levels of cDNA FLJ54111 and SERPINA3 were reduced in this group. Compared to the control group, patients with ATTR-CA exhibited higher levels of ceruloplasmin, SERPINA3, and VCAM1, as well as lower levels of ApoA-I, ApoA-II, clusterin, and gelsolin. Controls exhibited elevated levels of transthyretin and prothrombin. **Conclusions:** This study identified candidate serum biomarkers for diagnosing ATTR-CA and monitoring the effectiveness of tafamidis treatment.

## 1. Introduction

Cardiac transthyretin amyloidosis (ATTR-CA) is one of the most common forms of cardiac amyloidosis (CA). It occurs in two forms: hereditary (hATTR), which is caused by a variant of the transthyretin (TTR) gene, and wild-type (wtATTR), which is caused by abnormal folding of the TTR protein [1].

ATTR-CA is characterized by increased myocardial thickness on echocardiography and is often mistaken for other diseases involving myocardial thickening. Correct diagnosis is crucial, as treatment for ATTR-CA differs significantly from that for other diseases [2]. The introduction of ^99m^Technetium-3,3-diphosphono-1,2-propanodicarboxylic acid ({^99m^Tc}Tc-DPD) bone scintigraphy and the noninvasive diagnostic algorithm proposed by Gillmore et al. have made ATTR-CA diagnosis significantly less complicated [3]. Bone scintigraphy using {^99m^Tc}Tc-DPD has become a reliable technique for distinguishing ATTR-CA from other diseases. Nonetheless, this method has its limitations. First, it is not a commonly available diagnostic technique. Second, a lack of myocardial uptake of the radiopharmaceutical does not necessarily exclude ATTR-CA because certain specific variants of the TTR gene cause {^99m^Tc}Tc-DPD bone scintigraphy to exhibit very low sensitivity [4]. Therefore, innovative and more accessible tools are essential to enhance ATTR-CA diagnosis and make it more widely available. Identifying unique serum proteins associated with ATTR-CA could have significant diagnostic implications. In clinical practice, ATTR-CA is often diagnosed by differentiating it from other pathologies that cause myocardial thickening. However, no studies have compared the serum protein profiles of ATTR-CA patients with those of patients suspected of having CA, but for whom CA has been ruled out. Comparing the proteomes of ATTR-CA patients before specific treatment and during tafamidis therapy could reveal potential biomarkers for evaluating treatment efficacy. This study aimed to identify, through proteomic analysis, serum biomarkers that distinguish patients with ATTR-CA from those with increased myocardial thickness but without CA. The study also aimed to propose potential predictive biomarkers for monitoring the efficacy of tafamidis treatment.

## 2. Materials and Methods

The study follows the Declaration of Helsinki and received approval from the Institutional Ethics Committee of the National Institute of Cardiology (IK.NPIA.0021.30.1910/21). Written informed consent was obtained from all participants involved in the study.

### 2.1. Study Population

The study included consecutive patients with ATTR-CA, as well as patients exhibiting increased myocardial thickness and suspicion of cardiac amyloidosis (CA). These patients were ultimately excluded from the CA diagnosis and served as the control group. CA was suspected in patients with a left ventricular (LV) wall thickness of at least 12 mm and at least one red flag or clinical scenario, as previously detailed [1,5,6].

All patients underwent a comprehensive evaluation, including a detailed medical history, physical examination, blood tests, standard 12-lead electrocardiography, transthoracic echocardiography, and {^99m^Tc}Tc-DPD bone scintigraphy. Blood tests included measurements of N-terminal pro-B-type natriuretic peptide (NT-proBNP) and high-sensitivity cardiac troponin T (hs-cTnT), as well as free light chain testing and immunofixation of serum and urine to exclude light chain amyloidosis. Furthermore, patients with ATTR-CA underwent TTR gene sequencing.

Patients with ATTR-CA were divided into two groups: one group of patients who had not yet received specific treatment, and another group who had been treated with tafamidis for at least six months.

### 2.2. Blood Sampling

Venous blood samples were collected from the participants and centrifuged at 2000× *g* for 15 min at room temperature using a 5702R centrifuge (Eppendorf, Hamburg, Germany). The plasma was then aliquoted and stored at −80 °C using a TSE320VGP freezer (Thermo Fisher Scientific, Bremen, Germany) until analysis. Repeated freeze–thaw cycles were avoided.

### 2.3. Principle

Sample preparation involved several sequential steps: albumin and IgG depletion from serum, denaturation, reduction, alkylation, tryptic digestion, and desalting with solid-phase extraction (SPE), followed by evaporation and dissolution of the sample. During analysis, repeated freeze–thaw cycles of the samples were avoided. High-resolution mass spectrometry with a data-dependent acquisition (DDA) method was employed to analyze the tryptic digestion using liquid chromatography–tandem mass spectrometry (LC-MS/MS).

### 2.4. Depletion (Albumin/IgG Removal)

Albumin and IgG depletion were carried out using an Albumin/IgG Removal Kit (Thermo Fisher Scientific, Bremen, Germany). Briefly, 170 µL of gel slurry was transferred to each spin column (Sigma-Aldrich, Darmstadt, Germany), and the columns were centrifuged at 10,000× *g* for one minute. After discarding the buffer, a bottom plug was inserted into each column, which was then placed into a labeled LoBind tube (Eppendorf, Hamburg, Germany). Then, 10 µL of the serum sample and 50 µL of the bind/wash buffer were added to each column. The columns were then capped from the top and vortexed to form a suspension. The samples were incubated for 10 min at room temperature on an orbital shaker at a mixing speed sufficient to keep the mixture suspended. After incubation, the bottom plug was removed, and the column was centrifuged at 10,000× *g* for 1 min. The recovered filtrate was reapplied to the resin bed for another round of incubation and shaking. The bottom plug was removed from the column, and the cap was loosened. The column was placed in a tube and centrifuged at 10,000× *g* for 1 min to collect the filtrate. Then, 75 µL of binding/wash buffer was added to the gel bed, and the column was centrifuged at 10,000× *g* for 1 min to collect the wash in the same tube as the filtrate from the previous step. The flow-through was collected for further processing.

### 2.5. Sample Preparation

The depleted samples were denatured using a solution containing 12.5 µL of 8 M guanidine hydrochloride (GnHCl, Glentham Life Sciences, Corsham, UK), 12.5 µL of 100 mM Tris-HCl, and 5 µL of 50 mM DTT DL-dithiothreitol (Sigma-Aldrich, Darmstadt, Germany). The samples were then incubated at 60 °C for 15 min. Next, the samples were alkylated in the dark with 5 µL of 100 mM iodoacetamide (IAA) (Sigma-Aldrich, Darmstadt, Germany), after which they were diluted with 40 µL of 100 mM Tris-HCl (Merck, Darmstadt, Germany). The samples were digested with 10 µL of trypsin (G-Biosciences, Newark, DE, USA) at 37 °C for 17 h. Digestion was quenched with 5 µL of 50% formic acid (FA) (Merck, Darmstadt, Germany). Desalting was performed using an Attract SPE^®^ C18 disk plate (Affinisep, Normandy, France). The disk was conditioned with 500 µL of methanol (VWR Chemicals, Leicestershire, UK), centrifuged at 800× *g*, equilibrated with 500 µL of mixture B (80% acetonitrile (ACN) with 0.5% acetic acid (*v*/*v*)) and centrifuged at 800× *g*, and then with mixture A (water with 0.5% (*v*/*v*) acetic acid) and centrifuged at 800× *g*. The digested samples were applied to the disk and washed with 500 µL of mixture A, then centrifuged at 800× *g*. The disk was then eluted with 500 µL of mixture B, and the eluates were transferred to vials. The samples were evaporated using an Ultravap Levante (Porvair Sciences, Wales, UK) at 60 °C for 45 min. The dried peptides were reconstituted in 500 µL of 5% ACN with 0.1% FA before LC-MS/MS analysis.

### 2.6. LC-MS/MS Analysis

The samples were analyzed in trap-elute mode using an M5 Micro LC-TE system and a ZenoTOF 7600 mass spectrometer equipped with an OptiFlow ESI ion source (Sciex, Framingham, MA, USA). Chromatographic separation was performed using a nano Ease M/Z Symmetry C18 trap column (100 Å, 5 µm, 300 µm × 25 mm) and a nano Ease M/Z Peptide CSH C18 analytical column (130 Å, 1.7 µm, 300 µm × 150 mm) (Waters, Milford, MA, USA). The trap column was kept at room temperature, while the analytical column was maintained at 50 °C. The injection volume was 5 µL, and the mobile phases were A (water with 0.1% FA) and B (ACN, with 0.1% FA). The sample was injected at a flow rate of 5 µL/s. The trap loading flow rate was set at 50 µL/min, and the analytical loading flow rate was set at 7 µL/min, according to the flow gradient. The trapping gradient program begins at 0 min with B at 5% and A at 95%. After 1.5 min, the analytical gradient begins. During the analytical gradient, the trap column undergoes a series of gradient changes from A to B and back again to wash it before the next run. The analytical gradient flow rate was maintained at 7 µL/min during peptide elution. The gradient program begins with B at 5% and A at 95%, rising to 10% B after one minute. Then it rises to 45% in the 25th minute. From 25.1 to 30 min, the column is washed at 98% with a flow rate of 10 µL/min. From 30.1 min on, the column equilibrates at 5%, ending at the conclusion of the method at 36.5 min.

MS and MS/MS data were acquired in positive ionization data-dependent acquisition (DDA) mode, covering a mass range of 300–1800 *m*/*z* in TOF-MS scanning and 50–2000 *m*/*z* in TOF-MS/MS scanning. The accumulation times were 0.2 s and 0.01 s, respectively. Zeno trapping was enabled for all MS/MS experiments. The DDA criteria included the following: 50 maximum candidate ions, an intensity threshold of 150 cps, dynamic collision energy, a charge state from 2 to 5, a mass tolerance of 10 ppm, and exclusion of former target ions for 5 s after two occurrences. The analysis lasted 34 min, with a total scan time of 1.02 s and approximately 1999 cycles. The declustering potential (DP) was set to 80 V, and the source parameters were set as follows: gas (GS1) at 20 psi, Gas 2 (GS2) at 40 psi with a temperature of 400 °C, curtain gas (CUR) at 45 psi, and spray voltage at 5000 V.

### 2.7. Data Analysis

A qualitative proteomic analysis was performed. The protein profile was compared between the following groups:

- the ATTR-CA group and the control group;
- ATTR-CA patients before and after receiving at least six months of tafamidis treatment.

Identification and localization of selected post-translational modifications (PTMs) were performed using PEAKS 12 software (Dell/Bioinformatics Solutions Inc., Waterloo, ON, Canada) based on the accurate measurement of the masses of tryptic peptides from precursor ions (TOF-MS) and their corresponding fragmentation patterns (TOF-MS/MS). The MS and MS/MS data from all samples were aligned by retention time (RT) and searched against the human protein sequence database (UniProtKB, ID 9606, 30 September 2024, 204,500 entries). Only significant matches, determined by parallel statistical analysis, were considered identified.

### 2.8. Principal Component Analysis (PCA)

The peptide area table of the identified proteins in the samples was generated using PEAKS 12 software (Bioinformatics Solutions Inc., Waterloo, ON, Canada) and then imported into MarkerView 1.4 software (Sciex, Framingham, MA, USA) for PCA analysis. Peptide peak areas were normalized using the most likely ratio (MLR) method [7,8,9]. The MLR method works best for normally distributed data, assuming that the majority of analytes do not change or have similar abundances and that no internal standards were used. The normalized data were subjected to principal component discriminant analysis (PCA-DA), and the data were divided into three groups. Pareto scaling with square root weighting was applied to graphically present the PCA scores and loadings. The groups included in the analysis did not have an equal number of participants. Therefore, Welch’s *t*-test was applied to identify statistically significant peptides that distinguish each group from the others. In each test, the data were classified by *t*-values, and signals with highly positive or negative correlations between the tested group and the other groups, and the *p*-value < 0.05 was considered a potential marker for the discrimination of groups.

### 2.9. Statistical Analysis

The data processing phase included protein identification and localization of selected post-translational modifications (PTMs), as well as sample profiling and statistical comparison using PCA and the two-sample *t*-test. These tests were performed with and without false discovery rate (FDR) correction for multiple comparisons. Welch’s *t*-test, which is suitable for groups with unequal sample sizes and variances, was applied to identify significant differences between the two groups in the “top ten” results. The focus was on the “top ten” variables because analysis of more than 18,000 data inputs revealed that differences only become apparent after weighting the variables (x^2^ or log). Weighted discriminant analysis highlighted subtle differences that would not be noticeable with standard methods. The results of the Welch’s *t*-test confirmed that using the correct methodology is essential for identifying significant signals in large datasets.

The demographic and clinical characteristics of the study cohort were compared using the MedCalc 12.1.4.0 software (MedCalc; Mariakerke, Belgium). After checking for normal distribution using the Shapiro–Wilk test, continuous variables were compared using Student’s *t*-test or the Mann–Whitney test, as appropriate. Continuous variables that were normally distributed were shown as mean {standard deviation}, and continuous variables that were not normally distributed were shown as median {interquartile range}. Categorical variables were expressed as frequencies (percentages) of patients and were compared using the chi-square (χ^2^) test or Fisher’s exact test. A two-sided *p*-value less than 0.05 was considered statistically significant.

## 3. Results

The study included 15 patients with ATTR-CA before receiving specific treatment, 11 patients with ATTR-CA who were treated with tafamidis for at least six months, and 13 patients with suspected but excluded cardiac amyloidosis (control group). All of the patients in the study were male. A wild-type form of the disease was diagnosed in all patients with ATTR-CA. Patients with ATTR-CA were older than the control group (75.3 years (±5.5) vs. 67.8 years (±10.4), *p* = 0.008). Electrocardiogram (ECG) findings characteristic of CA, such as a pseudoinfarct pattern or low QRS voltage, were more frequently observed in ATTR-CA patients than in the control group (*p* = 0.005 and *p* = 0.03, respectively). There were no statistically significant differences between the groups in terms of New York Heart Association (NYHA) class or comorbidities such as coronary artery disease, diabetes mellitus, or atrial fibrillation. However, arterial hypertension was more prevalent in the control group (*p* = 0.03). Echocardiography revealed that patients with ATTR-CA had greater maximum and posterior wall thicknesses (20.0 mm {18.0–23.0} vs. 17.0 mm {14.0–20.0}, *p* = 0.01 and 16.0 mm {16.0–18.75} vs. 12.0 mm {11.5–13.0}, *p* < 0.001, respectively). Additionally, cardiac biomarkers levels of NT-proBNP and hs-cTnT were higher in ATTR-CA patients (2645 pg/mL {1426.25–3778.5} vs. 299.5 pg/mL {226.0–932.5}, *p* < 0.001 and 59.0 ng/L {38.25–85.25} vs. 17.0 ng/L {13.25–23.0}, *p* < 0.001, respectively).

The results of the comparative proteomic analysis among the study groups are displayed in Table 1, Table 2 and Table 3, which show significant alterations in protein levels across the different groups. Statistical analysis revealed notable differences, with *t*-values ranging from −8.38 to 4.05, and *p*-values of less than 0.01 for most comparisons. These results provide strong evidence of differences in the mean values among the groups.

A comparative analysis of the control group and patients with ATTR-CA revealed reduced levels of gelsolin, alpha-1-antitrypsin, vascular cell adhesion molecule one isoform A variant (VCAM1), ceruloplasmin, and clusterin. Several proteins showed notable increases in the control group, including prothrombin, TTR, apolipoprotein A-I (ApoA-I), apolipoprotein A-II (ApoA-II), and phosphatidylcholine-sterol acyltransferase (LCAT).

In patients with ATTR-CA prior to specific treatment, prothrombin concentrations were lower. Furthermore, this group exhibited reduced levels of transferrin, dopamine beta-hydroxylase, monocyte differentiation antigen CD14, and alpha-2-macroglobulin. Conversely, increased concentrations were observed for ceruloplasmin, apolipoprotein E, serpin family A member 1, and cDNA FLJ54111, which is highly similar to serotransferrin.

In contrast, the ATTR-CA group treated with tafamidis showed reduced levels of cDNA FLJ54111, which is highly similar to serotransferrin and SERPINA3. Levels of several proteins were elevated in this group, including epididymis secretory sperm binding protein Li 44a (SERPINA1), paraoxonase 1, and complement C2.

**Figure 1:**
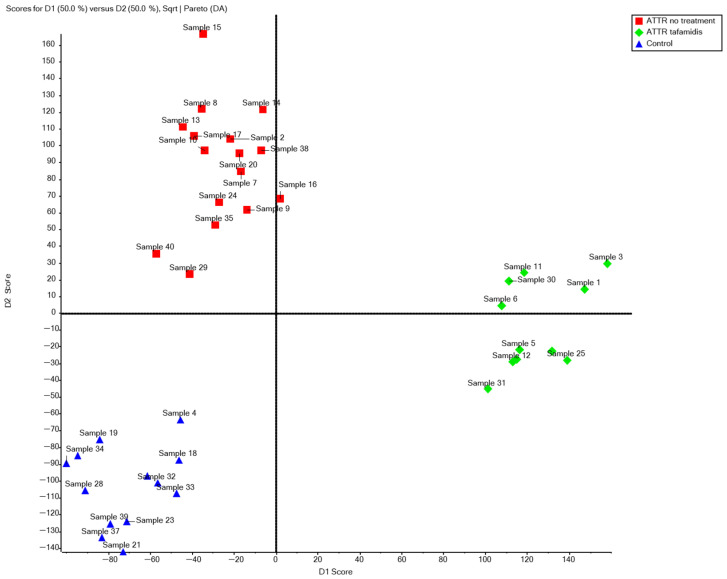
Analysis of area data for peptides assigned to identified proteins.

Many proteins did not show statistically significant differences between groups. However, this should not be directly interpreted as evidence of their biological stability. These results may reflect individual variability or methodological factors. Without repeated measurements or biological replicates, subtle or subject-specific variations may go undetected. Analysis of the area under the chromatographic peak of the peptide data for peptides assigned to identified proteins (Figure 1) revealed significant differences in the distribution of results between samples. Figure 1 shows that samples from ATTR-CA patients treated with tafamidis had higher D1 values than those from untreated ATTR-CA patients. This suggests that treatment positively impacts peptide signal intensity. Additionally, outlier points highlight samples with unusually high results that merit further investigation.

## 4. Discussion

Proteomic analysis is an effective technique for identifying new biomarkers and improving our understanding of disease pathophysiology [10,11,12,13,14]. During the APOLLO study, proteomic analysis of patients with hATTR and polyneuropathy identified a new biomarker: the neurofilament light chain (NfL). NfL may enable earlier diagnosis of patients with hATTR polyneuropathy and assist in monitoring disease progression [10,11,14].

Another study compared the serum protein profiles of patients with hATTR cardiomyopathy (n = 8) and hATTR polyneuropathy (n = 8) to those of the control group (n = 10) using proteomic analysis [12]. Although the study was conducted on a small number of patients, it revealed 18 protein level variations unique to hATTR cardiomyopathy, including significantly lower TTR levels in this group [12].

In a subsequent study, the serum proteomes of several patients with hATTR (n = 8) and wtATTR (n = 10) were compared to healthy volunteers (n = 10) [13]. Significant differences in proteomic profiles were found between ATTR patients’ serum samples and control samples. Furthermore, 27 protein-level variations exclusive to wtATTR were identified, including reduced prothrombin and fibrinogen alpha chain levels, as well as elevated SERPINA3, VCAM1, and ceruloplasmin concentrations.

Our research, which was intended as a pilot study, aimed to compare the proteomic profiles of patients with increased myocardial thickness and suspicion of CA, in whom CA was ultimately excluded, to those with ATTR-CA before and after tafamidis treatment. The objective was to identify potential biomarkers that could differentiate between these groups and serve as potential biomarkers for ATTR-CA while providing information on the effects of treatment.

Our results, which are consistent with previous data, show elevated levels of TTR and prothrombin in the control group [12,13]. As in previous reports, patients with ATTR-CA had more elevated levels of SERPINA3, VCAM1, and ceruloplasmin than controls [13]. Additionally, we observed variations in protein levels between ATTR-CA patients before and after tafamidis treatment. SERPINA1 levels were reduced in the ATTR-CA group before a particular treatment and elevated in the ATTR-CA group treated with tafamidis. In contrast, levels of cDNA FLJ54111 highly similar to serotransferrin were elevated in the ATTR-CA group before a specific treatment and reduced in the ATTR-CA group treated with tafamidis.

After validation, proteins with observed differences in levels between the study groups could potentially act as ATTR-CA biomarkers. These proteins have various functions and play roles in multiple processes. SERPINA3, also known as alpha-1-antichymotrypsin, is a serine protease inhibitor that helps regulate protease activity and inflammation [14]. Differences in SERPINA3 levels between ATTR-CA patients and controls may indicate the modulation of inflammation. ApoA-I, the main protein component of high-density lipoprotein (HDL), plays a crucial role in cholesterol transport and cardiovascular protection [15]. Elevated ApoA-I levels were observed in the control group and in ATTR-CA patients before receiving specific treatment. However, these levels decreased in ATTR-CA patients treated with tafamidis. This change may indicate alterations in lipid metabolism and improvements in cardiovascular health due to tafamidis treatment. Another HDL component involved in lipid metabolism and cholesterol transport, ApoAII, was elevated in the control group but exhibited a negative correlation in ATTR-CA patients before receiving specific treatment [16,17]. TTR is a transport protein that carries thyroxine and retinol-binding proteins [18,19]. It serves as the precursor protein in ATTR. Previous studies have shown that patients with hATTR cardiomyopathy have significantly lower TTR levels than patients with wtATTR or hATTR polyneuropathy. Changes in the cDNA FLJ54111, highly similar to serotransferrin, may reflect alterations in iron metabolism due to treatment with tafamidis. Our dataset identified multiple serotransferrin isoforms, including the canonical form (P02787) and cDNA FLJ54111, highly similar to serotransferrin. Although the latter lacks the N-terminal region and exhibits a single amino acid substitution (Ser150 instead of Gly), it was included in the final identification due to the presence of unique peptides that distinguish it from other serotransferrin isoforms. These isoform-specific peptides (listed in Table 1) enable reliable discrimination despite the high overall sequence similarity. This justifies the separate annotation of FLJ54111 alongside other serotransferrins. Its inclusion reflects the sensitivity of the proteomic analysis and its ability to resolve even highly similar sequence variants. Further analysis of these proteins may provide valuable insight into the biological effects of tafamidis treatment in patients with ATTR-CA. Carboxylic ester hydrolase is crucial for drug metabolism and lipid degradation because it is involved in the hydrolysis of ester bonds [20,21,22]. Lower levels of this protein in ATTR-CA patients before treatment may indicate altered metabolism and detoxification processes. Dopamine beta-hydroxylase, responsible for converting dopamine to norepinephrine, plays a crucial role in the function of the sympathetic nervous system. The observed differences may reflect changes in neurohormonal regulation related to ATTR and its treatment. The presence of transferrin and cDNA FLJ56687, highly similar to serotransferrin (an essential iron transport protein critical for iron homeostasis and cell respiration), may indicate altered iron metabolism, which could exacerbate amyloid pathology. The monocyte differentiation antigen CD14 acts as a coreceptor that detects bacterial lipopolysaccharides and activates the immune response. Variations in CD14 levels may indicate differences in immune activation and inflammation among different groups. Alpha-2-macroglobulin, a broad-spectrum protease inhibitor, regulates proteolytic activity and inflammation. In patients with ATTR-CA before receiving specific treatment, there is a negative correlation with this protein level, similar to what is observed in the control group. However, a positive correlation is noted in patients with ATTR-CA who are treated with tafamidis. This may suggest increased protease activity and inflammation. SERPINA1 helps protect tissues from enzyme degradation. Variations in SERPINA1 levels may reflect differences in protease inhibition and tissue protection mechanisms. Prothrombin, a precursor of thrombin, is a key enzyme in blood coagulation. Variations in prothrombin levels (a positive correlation in the control group and a negative correlation in ATTR-CA patients before specific treatment) may indicate changes in coagulation status and increased thrombotic event risk. Gelsolin, an actin-binding protein involved in cytoskeletal remodeling and inflammation, showed a negative correlation in the control group compared to ATTR patients [23]. Alpha-1-antitrypsin, a serine protease inhibitor that protects tissues from inflammatory cells, demonstrated a negative correlation in the control group compared to ATTR patients. VCAM-1 plays a crucial role in the adhesion of leukocytes to the vascular endothelium. It is significant in the inflammatory response and progression of cardiovascular diseases [24,25,26,27,28,29]. Ceruloplasmin is a copper-containing protein that plays a role in iron metabolism and has antioxidant properties. Differences in ceruloplasmin levels may indicate variations in oxidative stress and iron metabolism [30]. Clusterin is a multifunctional chaperone protein that is involved in extracellular protein quality control and the inhibition of amyloid aggregation. It demonstrated a negative correlation in the control group compared to ATTR patients, which suggests a potential compensatory response to transthyretin misfolding and deposition [31,32,33].

Proteomic analysis in our study revealed significant differences in protein levels between the groups. Our results and previous reports suggest the potential of several proteins as biomarkers for distinguishing ATTR-CA and for enhancing our understanding of the pathophysiological processes of this disease and its treatment effects in patients with ATTR-CA. Further studies are needed to verify the usefulness of prothrombin, SERPINA3, VCAM1, and ceruloplasmin as diagnostic biomarkers of ATTR-CA, as well as SERPINA1 and cDNA FLJ54111, which is highly similar to serotransferrin, as biomarkers for predicting the effects of ATTR-CA treatment.

## 5. Conclusions

The present study contributes to the growing understanding of cardiac transthyretin amyloidosis (ATTR-CA) by exploring potential serum biomarkers that can support diagnostic and therapeutic strategies. Incorporating a control group with increased myocardial thickness and excluding cardiac amyloidosis allowed the identification of proteomic changes that may be characteristic of ATTR-CA. Observations from proteomic analysis suggest that tafamidis treatment may be associated with alterations in proteins involved in lipid metabolism, iron transport, neurohormonal regulation, and immune response. In contrast, differences in patients with protein profiles in untreated ATTR-CA provide additional information on the complex pathophysiology. These findings may offer preliminary evidence for potential biomarkers that could complement existing diagnostic tools and support the monitoring of the therapeutic response. However, given the exploratory nature of this study, validation in a larger population is needed to confirm the clinical relevance of the proposed biomarkers. Future studies could also provide a more comprehensive understanding of how these proteomic changes correlate with disease progression and treatment outcomes.

## Figures and Tables

**Table 1:** Comparative analysis of serum proteomic biomarkers in the control group versus patients with cardiac transthyretin amyloidosis.

Protein Symbol	Protein Name	*t*-Value	*p*-Value	Fold Change
Reduced levels				
A0A8V8TND7	Gelsolin	−4.24	<0.001	0.320
A7L8C6	Alpha-1-antitrypsin	−3.88	<0.001	0.070
G3V595	Serpin family A member 3 (SERPINA 3)	−2.67	0.01	0.527
Q53FL7	Vascular cell adhesion molecule 1 isoform a variant	−3.54	0.001	0.244
Q1L857	Ceruloplasmin	−3.51	0.001	0.250
A0A384NKS6	Clusterin	−3.25	0.003	0.080
Elevated levels				
P00734	Prothrombin	3.03	0.006	1.770
J3QKT0	Phosphatidylcholine-sterol acyltransferase (LCAT) (138 aa)	3.43	0.002	2.113
P04180	Phosphatidylcholine-sterol acyltransferase (LCAT) (440 aa)	3.85	<0.001	2.343
A6XGL1	Transthyretin (TTR)	4.05	<0.001	2.087
V9GYM3	Apolipoprotein A-II	3.63	0.001	1.787
A0A024R3E3	Apolipoprotein A-I	2.94	0.006	1.432
P05543	Thyroxine-binding globulin (SERPINA 7)	3.31	0.002	1.563

aa—amino acids.

**Table 2:** Comparative analysis of serum proteomic biomarkers in ATTR-CA patients before specific treatment versus ATTR-CA patients after at least 6 months of tafamidis treatment and the control group.

Protein Symbol	Protein Name	*t*-Value	*p*-Value	Fold Change
Reduced levels				
P01024	Complement C3	−2.56	0.01	0.513
P09172	Dopamine beta-hydroxylase	−2.55	0.001	0.219
C9JB55	Transferrin	−2.51	0.01	0.076
P08571	Monocyte differentiation antigen CD14	−2.90	0.007	0.213
P01023	Alpha-2-macroglobulin (1474 aa)	−2.49	0.01	0.212
P00734	Prothrombin	−2.54	0.01	0.664
Elevated levels				
A0A024R3E3	Apolipoprotein A-I	2.47	0.01	1.949
F5H1E8	Alpha-2-macroglobulin (90 aa)	2.32	0.02	2.277
Q1L857	Ceruloplasmin	2.73	0.01	7.166
E7ERP7	Apolipoprotein E	2.59	0.02	5.561
B4DI57	cDNA FLJ54111, highly similar to Serotransferrin	2.99	0.008	6.426
A0A0G2JRN3	Serpin family A member 1	2.50	0.02	12.463
P0DOX7	Immunoglobulin kappa light chain	2.66	0.01	1.887

aa—amino acids; ATTR-CA—cardiac transthyretin amyloidosis.

**Table 3:** Comparative analysis of serum proteomic biomarkers in ATTR-CA patients after at least six months of tafamidis treatment versus ATTR-CA patients before specific treatment and the control group.

Protein Symbol	Protein Name	*t*-Value	*p*-Value	Fold Change
Reduced levels				
G3V595	Serpin family A member 3 (SERPINA 3)	−6.35	<0.001	0.057
A6XGL1	Transthyretin	−8.38	<0.001	0.004
A0A024R3E3	Apolipoprotein A-I	−3.31	0.002	0.305
B4DI57	cDNA FLJ54111, highly similar to Serotransferrin	2.64	0.01	1.729
Q5VY30	Retinol-binding protein	−3.60	<0.001	0.296
Elevated levels				
A8K2N0	cDNA FLJ77835, highly similar to Homo sapiens complement component 1, s subcomponent (C1S), transcript variant 2, mRNA	2.70	0.01	12.281
Q13784	Apolipoprotein A-IV	2.64	0.01	2.375
B2R8I2	Histidine-rich glycoprotein	2.63	0.02	14.880
P01023	Alpha-2-macroglobulin	2.17	0.04	2.044
E9KL23	Epididymis secretory sperm binding protein Li 44a (SERPINA 1)	2.21	0.04	14.433
B1PWC6	Paraoxonase 1	2.38	0.03	8.949
A0A1U9X8X9	Complement C2	2.11	0.05	5.886
V9HW68	Epididymis luminal protein 214	2.03	0.06	1.694
Q53FV4	Lumican	2.34	0.03	2.152

aa—amino acids; ATTR-CA—cardiac transthyretin amyloidosis.

## Data Availability

The original contributions presented in this study are included in the article. Further inquiries can be directed to the corresponding author.
